# Correlation of NO and ET-1 Levels with Blood Pressure Changes in Hemodialysis Patients after Arteriovenous Fistula Surgery

**DOI:** 10.3389/fsurg.2022.905372

**Published:** 2022-05-16

**Authors:** Yanli Li, Hanxiao Lu, Yu Sun

**Affiliations:** Hemodialysis Room, The First Affiliated Hospital of Qiqihar Medical College, Qiqihar, China

**Keywords:** arteriovenous fistula surgery, hemodialysis, blood pressure changes, NO, ET-1

## Abstract

Hemodialysis (HD) is the most common renal replacement therapy for patients with end-stage renal disease (ESRD) and can significantly reduce mortality and improve the quality of life of patients. The occurrence of intradialytic hypotension and intradialytic hypertension are important risk factors for death and disability during dialysis in patients with ESRD, yet their etiology remains unclear, and some studies suggest that nitric oxide (NO) and endothelin-1 (ET-1) may play an important role in these hemodynamic alterations. For this purpose we examined the changes in NO and ET-1 levels during hemodialysis in 30 patients on maintenance hemodialysis (MHD) after arteriovenous fistula surgery. Thirty dialysis patients were divided into group I (stable blood pressure during dialysis), group II (Intradialytic hypotension) and group III (Intradialytic hypertension) according to the change of blood pressure (BP) during hemodialysis, with 10 cases in each group. BP of MHD patients were measured Pre-dialysis (Pre-D), at 1 h of dialysis (1h-D), at 2 h of dialysis (Mid-D, 2h-D), at 3 h of dialysis (3h-D), and at the end of dialysis (Post-D); and blood samples were taken from the arterial end at Pre-D, Mid-D, and Post-D to measure NO and ET-1 levels. The results of the analysis showed that as dialysis proceeded and ended, the NO levels in the three groups gradually decreased, with significant differences compared with those before dialysis (*p* < 0.05); the ET-1 levels in group III gradually increased, with significant differences compared with those before dialysis (*p* < 0.05), while the increasing trend of ET-1 levels in group I and group II was not significant. The increasing trend of MAP in group I was not significant (*p* > 0.05); MAP in group II showed a gradual decrease and MAP in group III showed an increasing trend, and the difference between MAP after dialysis and before dialysis was significant (*p* < 0.05). Correlation analysis showed a significant positive correlation between ET-1 levels and MAP in Group III at Mid-D (*r* = 0.847, *p* = 0.002). This shows that serum ET-1 and NO levels are significantly higher than normal in MHD patients after arteriovenous endovascular fistula surgery, and both ET-1 and NO levels are changing during dialysis, and there may be a link between their changes and blood pressure changes. It is suggested that the blood pressure fluctuations that occur during dialysis in MHD patients may be related to endothelial cell dysfunction.

## Introduction

Hemodialysis (HD) was clinically applied in the 1960s and is currently the most commonly used blood purification therapy for the treatment of end-stage renal disease (ESRD) (1, 2). In recent years, the composition ratio of dialysis complications has changed with the advancement of HD technology, including the improvement of dialysis machines, the improvement of dialysis water quality, the application of dialyzers with good membrane biocompatibility, etc. (3, 4). However, acute hemodynamic changes during dialysis are still common, mainly in the form of intradialytic hypotension and intradialytic hypertension (5, 6). Poor BP (BP) control is a common complication in patients with ESRD-HD and an independent risk factor for patient prognosis. Unlike the control of BP in general patients, HD patients need to control BP not only during HD but also during the fluctuation of BP during HD. However, some studies (6, 7) have shown that intra-dialysis hypotension and intra-dialysis hypertension caused by fluctuations in BP during dialysis can increase the incidence of cardiovascular time and mortality in HD patients. Therefore, the pathogenesis of hypotension and hypertension during HD, if fully understood and effectively controlled, may have a significant impact on the long-term survival and quality of life of HD patients.

Studies (8, 9) have found that endothelin (ET-1) and nitric oxide (NO) may be involved in the onset and progression of BP changes during dialysis in HD patients. ET-1 is the most potent vasoconstrictor substance identified, and it is widely distributed in cardiovascular and neural tissues, and the increased peripheral vascular resistance in essential hypertension may be related to its excessive production (10). NO is a gaseous biological messenger molecule that is widely involved in a variety of physiological and pathological processes in the body, and is considered to be a regulatory mediator in the regulation of vascular tone and maintenance of BP stability (11, 12). The aim of this study was to investigate the relationship between the expression of serum ET-1 and NO levels and BP changes in patients with different degrees of BP in ESRD treated with HD after action endovenous fistula surgery and their correlation.

## Materials and Methods

### Study Subjects

30 patients who underwent regular hemodialysis after arteriovenous endovascular fistula in our hemodialysis center from January 2020 to December 2021 were selected. Medical history, physical examination, X-ray examination, electrocardiogram, echocardiogram, and relevant laboratory tests were taken at the time of enrollment. A mercury column sphygmomanometer was used to measure BP in the right upper arm of patients, and 30 patients with ESRD were divided into three groups according to the changes in BP during HD, with 10 patients in each group: patients with stable BP during HD were group I; patients with hypotension during HD were group II, mainly manifested by a sudden drop in systolic blood pressure (SBP) with symptoms, SBP <90 mmHg or SBP drop ≥20 mmHg, or at least 50% of the dialysis treatment process showed systolic blood pressure <90 mmHg or SBP decrease ≥20 mmHg; hypertension during HD was the Group III, which mainly showed normal blood pressure or hypertension before dialysis, but the mean arterial pressure (MAP) increased more than 15 mmHg during dialysis compared with that before dialysis.

### Inclusion Criteria

Patients with ESRD aged >18 years, dialysis age >3 months, 2–3 dialysis sessions per week, 3.5–4 h per session. Combined with sleep apnea syndrome, insulin resistance syndrome and other diseases that more significantly affect ET-1 and NO levels.

### Exclusion Criteria

Patients with tumors; patients with failed kidney transplants; patients with abnormal mental behavior; patients with 2 interruptions or transfers from dialysis treatment; patients with acute complications such as infections and bleeding.

### Research Methodology

#### Treatment

All MHD patients were dialyzed 2 to 3 times a week for 4 h each time with Baxter CTl90 dialyzer, Fresenius 4008B or 4008S dialysis machine with blood flow rate of 250 mL/min and dialysate flow rate of 500 mL/min, normal heparin anticoagulation and bicarbonate dialysis.

#### Clinical Data were Collected

Including age, sex, primary disease, BP, dialysis time, previous cardiovascular disease, and biochemical indexes [included white blood cells (WBC), hemoglobin (Hb), platelets (PLT), serum albumin (SAB), triglycerides (TG), total cholesterol (TC), high-density lipoprotein (HDL), low-density lipoprotein (LDL), and parathyroid hormone (PTH)]. In ESRD patients, the age of dialysis (months), type of dialyzer used, ultrafiltration volume, BP Pre-dialysis (Pre-D), BP at l, 2, and 3 h of dialysis, and BP at the end of dialysis (Post-D) were recorded, and the brachial BP on the non-endovascular side was measured. Each measurement was taken 3 times and the mean value was taken.

#### Blood Specimen Collection

The blood specimens were collected at Pre-D, 2 h of dialysis (Mid-D, 2h-D) and at Post-D, respectively, by lowering the blood flow rate to 50 ml/min and taking blood specimens at the arterial end of the dialysis line after stopping ultrafiltration and ineffective dialysis for 1 to 2 min.

#### Blood Pressure Measured and Recorded

Patients in both groups were measured by dedicated medical staff with an electronic sphygmomanometer in a lying position with the measuring arm at the same level as the heart and the sphygmomanometer. 3 groups of dialysis patients rested for 10 min before dialysis, and the blood pressure was measured Pre-D, at 1 h of dialysis (1 h-D), at 2 h-D, at 3 h of dialysis (3h-D) and at Post-D on the upper arm without fistula. MAP = (SBP - diastolic blood pressure)/3 + diastolic blood pressure.

#### Laboratory Tests

blood samples were drawn from the arterial end of HD patients at Pre-D, at Mid-D and at Post-D. The detection of NO was performed by chemiluminescence method, and the kit was purchased from Nanjing Jiancheng Institute of Biological Engineering; the detection of ET-l was performed by enzyme-linked immunoassay, and the kit was purchased from ADL, USA. The reference range of normal value of NO: (39.00 ± 11.00) μmol/L; the reference range of normal value of ET-1: (50.8 ± 7.58) pg/mL.

### Statistical Methods

SPSS 22.0 statistical software was used to analyze the data, Prism 7.0 was used to create the statistical graphs. Count data were expressed as rates (%) with a chi-square test. The measurement data were described as mean ± standard deviation (Mean, SD) using t or F test. correlation between ET-1 and MAP was analyzed using pearson correlation analysis. *α* = 0.05 was the test level.

## Results

### Comparison of General Data Between HD Different BP Patient Groups

No statistically significant differences (*p* > 0.05) were found between group I, group II and group III in terms of general information such as gender, age, dialysis age, dry weight, water loss and primary disease (Table 1).

**Table 1:** Comparison of general data between HD different BP patient groups.

Information		Group I (*n* = 10)	Group II (*n* = 10)	Group III (*n* = 10)	*p* value
Male (%)		5 (50.00)	4 (40.00)	6 (60.00)	0.670
Age (years; Mean, SD)		65.49 ± 10.21	66.00 ± 8.51	66.33 ± 7.24	0.977
Dialysis age (months; Mean, SD)		60.46 ± 26.34	51.23 ± 25.20	46.35 ± 20.16	0.665
Dry weight (kg; Mean, SD)		62.81 ± 9.80	59.76 ± 7.62	58.77 ± 10.20	0.603
Water loss (kg)		2.56 ± 1.01	2.62 ± 0.33	2.45 ± 0.87	0.889
Primary disease	Diabetes mellitus (%)	0 (0.00)	1 (10.00)	4 (40.00)	0.185
	Chronic nephritis (%)	6 (60.00)	7 (70.00)	2 (20.00)	
	Hypertensive nephropathy (%)	2 (20.00)	1 (10.00)	2 (20.00)	
	Other (%)	2 (20.00)	1 (10.00)	2 (20.00)	

### Comparison of Clinical and Biochemical Indexes Among Group I, Group II and Group III

**Figure 1:**
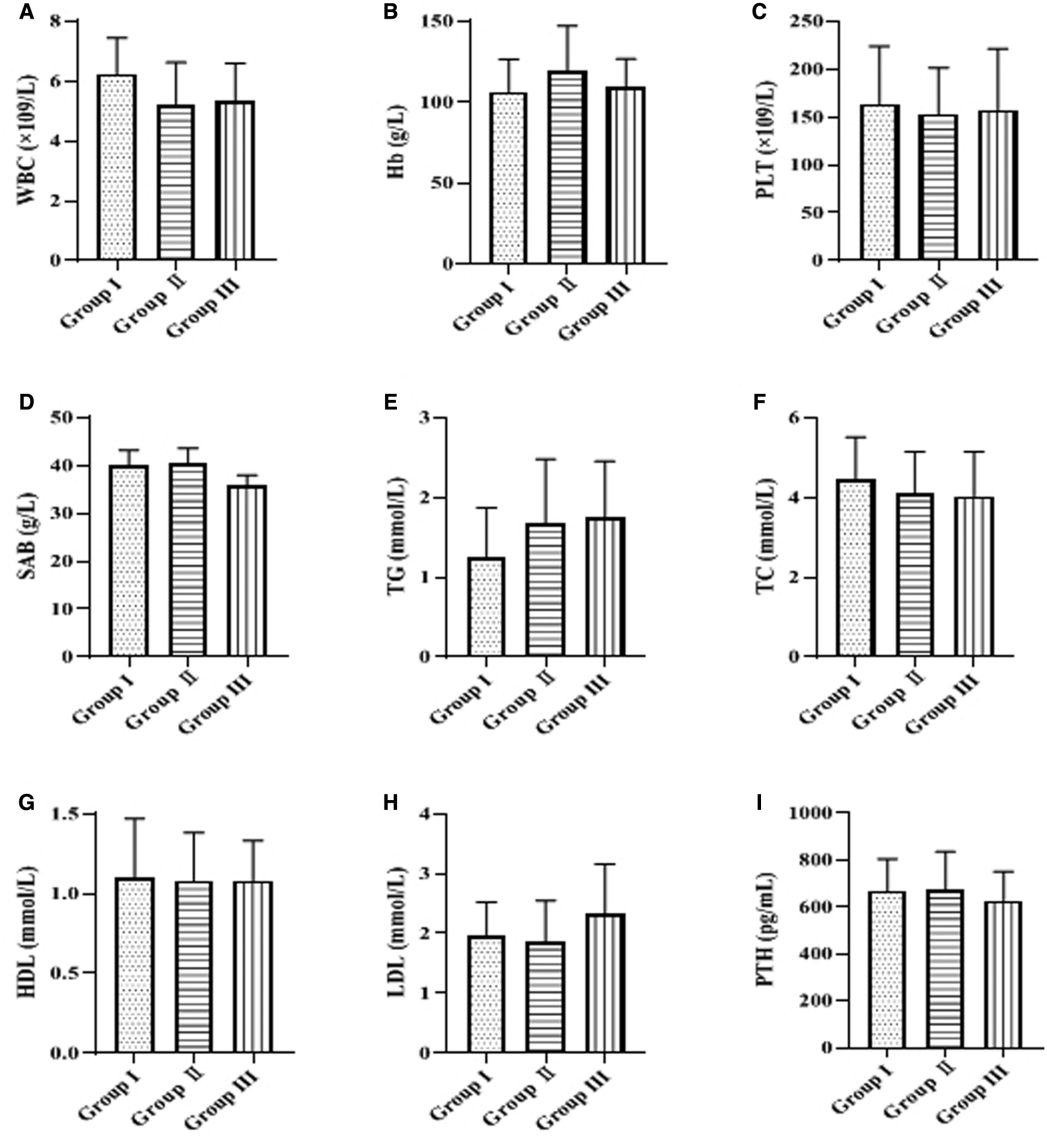
Comparison of clinical and biochemical indexes. The levels of white blood cells (WBC), hemoglobin (Hb), platelets (PLT), serum albumin (SAB), triglycerides (TG), total cholesterol (TC), high-density lipoprotein (HDL), low-density lipoprotein (LDL), and parathyroid hormone (PTH) are indicated in descending order from **A** to **I**.

Baseline values of serological markers such as WBC, Hb, PLT, SAB, TG, TC, HDL, LDL, and PTH were collected for comparison in the three groups. The results showed that none of the differences in clinical biochemical indices among group I, group II and group III were statistically significant (*p* > 0.05) (Figure 1).

### Comparison of Serum NO and ET-1 Levels at Pre-D, Mid-D and Post-D in Group I, Group II and Group III

**Figure 2:**
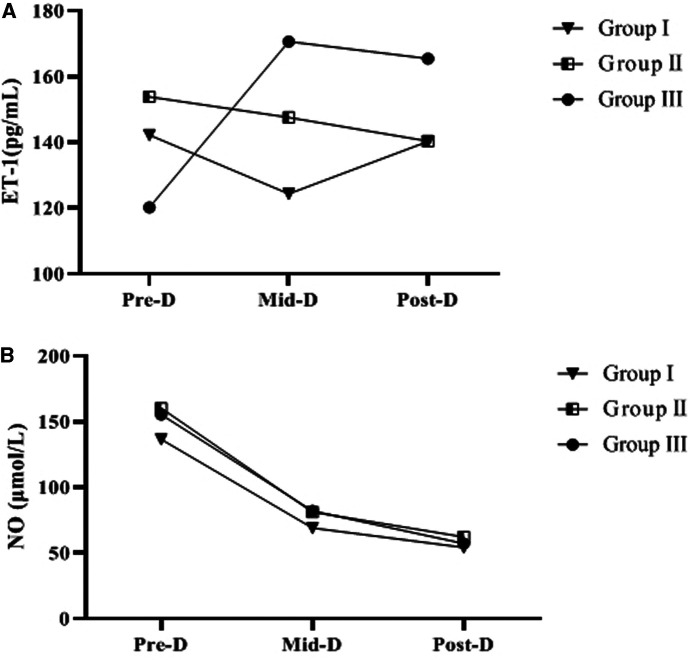
Trends of ET-1, NO levels in the three groups. **A** indicates the trend in ET-1 levels. **B** is the change trend of NO level.

Serum ET-1 and NO levels at Pre-D were significantly higher than normal in all three groups. Univariate analysis of ET-1 and NO levels Pre-D in the three groups showed that the differences were not statistically significant (*p* > 0.05). As dialysis progressed and ended, the NO levels in the three groups gradually decreased and were significantly different from those Pre-D (*p* < 0.05); the ET-1 levels in group III gradually increased and were significantly different from those Pre-D (*p* < 0.05), while the increasing trend of ET-1 levels in group I and group II was not significant (*p* > 0.05) (Table 2 and Figure 2).

**Table 2:** Comparison of serum NO and ET-1 levels at Pre-D, Mid-D and Post-D in group I, group II and group III (Mean, SD).

Indicators/groups	ET-1(pg/mL)	NO (μmol/L)
Group I (*n* = 10)		
Pre-D	142.26 ± 38.34	136.50 ± 40.81
Mid-D	124.37 ± 36.54	69.16 ± 20.28*
Post-D	140.30 ± 41.57	54.23 ± 8.04*^,^**
Group II (*n* = 10)		
Pre-D	153.86 ± 32.26	160.18 ± 43.65
Mid-D	147.60 ± 17.53	81.20 ± 25.48*
Post-D	140.34 ± 40.25	62.19 ± 17.20*
Group III (*n* = 10)		
Pre-D	120.23 ± 42.18	155.34 ± 46.71
Mid-D	170.62 ± 20.87*	82.06 ± 20.83*
Post-D	165.42 ± 44.58*	57.26 ± 10.2*^,^**

*Note: Compared with the same group at Pre-D*, *p < *0.05; compared with the same group at Mid-D,* **p < 0.05.

### Comparison of MAP During HD in Group I, Group II and Group III

**Figure 3:**
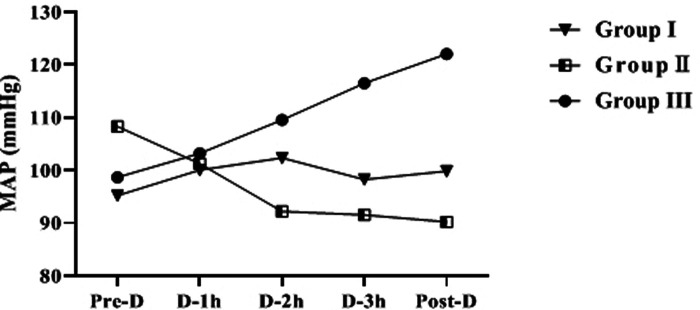
Trend of MAP changes during dialysis in 3 groups.

The difference of MAP at Pre-D in the three groups was not statistically significant (*p *> 0.05). As dialysis proceeded, the increasing trend of MAP in group I was not significant (*p* > 0.05); MAP in group II showed a gradually decreasing trend, and the MAP Post-D was significantly lower than that Pre-D (*p* < 0.05); MAP in group III showed an increasing trend, and the MAP Post-D was significantly higher than that Pre-D (*p* < 0.05) (Table 3 and Figure 3).

**Table 3:** Comparison of MAP during HD in group I, group II and group III (Mean, SD).

Time/group	Pre-D	1 h-D	2 h-D	3 h-D	Post-D
Group I (*n* = 10)	95.23 ± 12.28	100.06 ± 13.25	102.36 ± 11.58	98.27 ± 12.14	99.87 ± 10.56
Group II (*n* = 10)	108.30 ± 14.50	101.25 ± 12.77	93.23 ± 10.18	91.56 ± 10.26	90.24 ± 11.31*
Group III (*n* = 10)	98.70 ± 13.68	103.24 ± 15.16	109.58 ± 14.73	116.50 ± 13.29	122.06 ± 15.14*^,^**^,^***

*Note: Compared with the same group at Pre-D,* *p < 0.05; compared with group I at Post-D, **p < 0.05; compared with group II at Post-D, ***p < 0.05.

### Correlation Analysis of ET-1 Levels and MAP at 2h-D (Mid-D) in Group III

**Figure 4:**
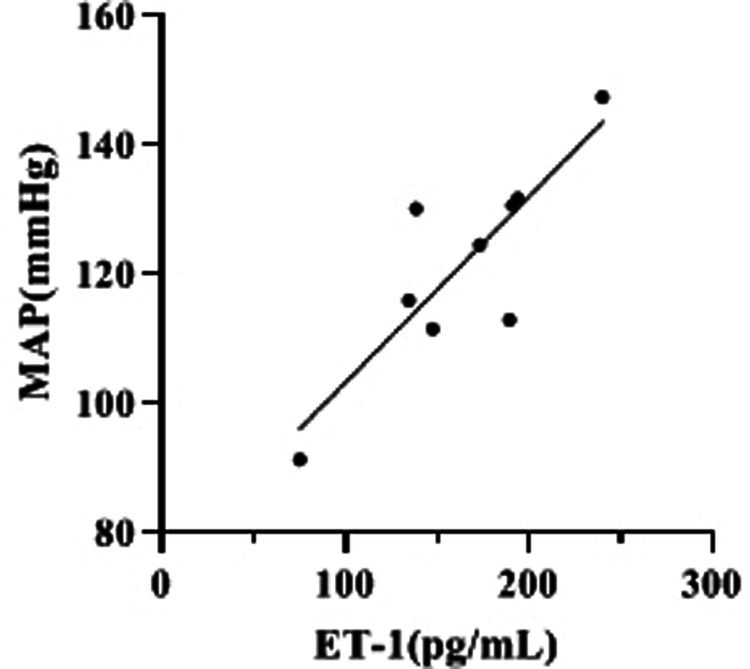
Correlation analysis of ET-1 levels and MAP at 2 h-D (Mid-D) in group III.

By compared and analyzed Figures 2, 3 we founded that both ET-1 levels and MAP levels showed an increasing trend during dialysis in group III. Therefore, we further analyzed the correlation between ET-1 and MAP in group III, and the showed that the level of ET-1 at Mid-D in group III was significantly positively correlated with MAP (*r* = 0.847, *p* = 0.002) (Figure 4).

## Discussion

With the improvement of socio-economic level and people's living conditions in China, more and more ESRD patients can choose continuous hemodialysis (MHD) as an alternative therapy. However, intradialytic hypotension and intradialytic hypertension due to fluctuations in BP during dialysis in patients with MHD are important risk factors for cardiovascular and cerebrovascular complications, morbidity and mortality, and all-cause mortality in dialysis patients (14, 15). Recent studies (16, 17) have shown that intradialytic hypotension is associated with patient prognosis and is an independent risk factor for high hospitalization rates in MHD patients, as well as reduced survival; intradialytic hypertension is an independent risk factor for increased mortality in MHD patients. There are numerous studies on the mechanisms of combined hypertension on dialysis, while the main mechanism for the occurrence of blood pressure fluctuations during dialysis is not clear (18). Meanwhile, the smooth control of blood pressure during dialysis in MHD patients has great significance for improving the prognosis of dialysis patients and improving the quality of survival of dialysis patients.

It is now believed that vascular endothelial injury is an important pathophysiological basis for cardiovascular disease, and in chronic kidney disease, endothelial dysfunction is often seen in the early stages of disease development, with patients commonly having abnormal endothelial function (19). Studies (20–22) have shown that vascular endothelial function is closely related to the dynamic levels of NO and ET-1, where ET-1 acts on ETA receptors in smooth muscle cells to promote calcium ion release and enhance extracellular calcium ion inward flow, resulting in strong vasoconstriction, which leads to an increase in SVR. NO is an important vasodilator with the opposite effect of ET-1, which is widely distributed in the body and can modulate vascular tone, inhibit platelet monthly endothelial adhesion and thrombosis to prevent vascular endothelial disorders and atherosclerosis (23, 24).

In this study, serum NO and ET-1 levels were found to be significantly higher than normal in patients with MHD, which is consistent with many literature reports (25–27). Meanwhile, this study found that serum NO levels in all three groups showed a gradual decrease during dialysis, and there were statistical differences between the three groups before and after dialysis when comparing serum NO levels themselves. The consideration may be related to the smaller molecular weight and stronger dispersion properties of NO, which is gradually removed by dialysis during the dialysis process and the amount removed increases with time, etc. However, we also found no statistical difference in NO levels in group II dialysis compared with the post-dialysis group, while the other two groups were statistically different, suggesting that the occurrence of hypotension in dialysis may still be related to NO levels. The effect of NO is not only to achieve peripheral vasodilation, but also to inhibit sympathetic nerve endings, to inhibit the release of catecholamines, and to inhibit their biological activity, which can inhibit autonomic function and can act synergistically with prostaglandins to cause a drop in blood pressure in patients on dialysis (28, 29). However, this study did not find any significant correlation between changes in NO levels before and after dialysis and changes in MAP, considering that the reason is mainly that NO, as a small molecule, can be removed by hemodialysis. In addition, this study also found that the changes in serum ET-1 levels at Pre-D and Post-D in group I and group II were not significant, but the ET-1 levels in group III were significantly higher at Mid-D than Pre-D and were still higher than the Pre-D levels at Post-D (*p* < 0.05), showing the same change trend as their MAP changes. The correlation analysis of MAP and ET-1 in dialysis showed a significant correlation (*p* < 0.05), which suggests that serum ET-1 levels may be associated with the development of hypertension in dialysis.

In conclusion, serum ET-1 and NO levels were significantly higher than normal in MHD patients after arteriovenous fistula surgery, and both ET-1 and NO levels were changing during dialysis, and there may be a link between their changes and blood pressure changes. suggesting that the blood pressure fluctuations that occur during dialysis in MHD patients may be related to endothelial cell dysfunction. As this study was a retrospective analysis, the conclusions obtained need to be supported by prospective studies with larger samples.

## Data Availability

The original contributions presented in the study are included in the article/Supplementary Material, further inquiries can be directed to the corresponding author/s.

## References

[1] Rastogi A Bhatt N Rossetti S Beto J. Management of hyperphosphatemia in end-stage renal disease: an new paradigm. J Ren Nutr. (2021) 31:21–34. 10.1053/j.jrn.2020.02.003 32386937

[2] Lipman ZM Yosipovitch G. An evaluation of difelikefalin as a treatment option for moderate-to-severe pruritus in end stage renal disease. Expert Opin Pharmacother. (2021) 22:549–55. 10.1080/14656566.2020.1849142 33190563

[3] Basile C Davenport A Mitra S Pal A Stamatialis D Chrysochou C Frontiers in hemodialysis: innovations and technological advances. Artif Organs. (2021) 45:175–82. 10.1111/aor.13798 32780472

[4] Pirklbauer M. Hemodialysis treatment in patients with severe electrolyte disorders: management of hyperkalemia and hyponatremia. Hemodial Int. (2020) 24:282–9. 10.1111/hdi.12845 32436307 PMC7496587

[5] Sars B van der Sande FM Kooman JP. Intradialytic hypotension: mechanisms and outcome. Blood Purif. (2020) 49:158–67. 10.1159/000503776 31851975 PMC7114908

[6] Kale G Mali M Bhangale A Somani J Jeloka T. Intradialytic hypertension increases non-access related hospitalization and mortality in maintenance hemodialysis patients. Indian J Nephrol. (2020) 30:85–90. 10.4103/ijn.IJN_153_19 32269431 PMC7132845

[7] Uchida M Kawano H Koga S Ikeda S Eishi K Maemura K. Ischemic heart disease cause of intradialytic hypertension in a patient with diabetic nephropathy. J Cardiol Cases. (2020) 22:181–3. 10.1016/j.jccase.2020.06.013 33014201 PMC7520537

[8] Rhee SY Song JK Hong SC Choi JW Jeon HJ Shin DH Intradialytic exercise improves physical function and reduces intradialytic hypotension and depression in hemodialysis patients. Korean J Intern Med. (2019) 34:588–98. 10.3904/kjim.2017.020 28838226 PMC6506736

[9] Tawfeek GA Kora MA Yassein YS Baghdadi AM Elzorkany KM. Association of pre-pro-endothelin gene polymorphism and serum endothelin-1 with intradialytic hypertension in an Egyptian population. Cytokine. (2021) 137:155293. 10.1016/j.cyto.2020.155293 33128922

[10] Zhang Y Zhang X Li J Liu X Cui C Yuan A Dry-weight reduction improves intradialytic hypertension only in patients with high predialytic blood pressure. Blood Press Monit. (2019) 24:185–90. 10.1097/MBP.0000000000000373 30807307

[11] Jenkins HN Rivera-Gonzalez O Gibert Y Speed JS. Endothelin-1 in the pathophysiology of obesity and insulin resistance. Obes Rev. (2020) 21:e13086. 10.1111/obr.13086 32627269 PMC7669671

[12] Sherlock LG Wright CJ Kinsella JP Delaney C. Inhaled nitric oxide use in neonates: balancing what is evidence-based and what is physiologically sound. Nitric Oxide. (2020) 95:12–6. 10.1016/j.niox.2019.12.001 31866361 PMC7594166

[13] Tenopoulou M Doulias PT. Endothelial nitric oxide synthase-derived nitric oxide in the regulation of metabolism. F1000Res. (2020) 9:F1000 Faculty Rev-1190. 10.12688/f1000research.19998.1 33042519 PMC7531049

[14] Fotiadou E Georgianos PI Chourdakis M Zebekakis PE Liakopoulos V. Eating during the hemodialysis session: a practice improving nutritional status or a risk factor for intradialytic hypotension and reduced dialysis adequacy? Nutrients. (2020) 12:1703. 10.3390/nu12061703 PMC7352512 32517256

[15] Theodorakopoulou M Loutradis C Bikos A Angeloudi E Schoina M Raptis V The effects of nebivolol and irbesartan on ambulatory aortic blood pressure and arterial stiffness in hemodialysis patients with intradialytic hypertension. Blood Purif. (2021) 50:73–83. 10.1159/000507913 33017836

[16] Chen KX. Academician kai-xian chen talks about the development of traditional chinese medicine and global medicine. World J Tradit Chin Med. (2020) 6:1–11. 10.4103/wjtcm.wjtcm_30_19

[17] Al-Said J Suyao C. Central systolic and diastolic blood pressure pressures during hemodialysis. Saudi J Kidney Dis Transpl. (2021) 32:170–3. 10.4103/1319-2442.318519 34145127

[18] Hartwig SV Hacon SS Oliveira BFA Jacobson LDSV Sousa RFV Ignotti E. The effect of ambient temperature on blood pressure of patients undergoing hemodialysis in the Pantanal-Brazil. Heliyon. (2021) 7:e07348. 10.1016/j.heliyon.2021.e07348 34235283 PMC8246300

[19] Okada H Yoshida S Hara A Ogura S Tomita H. Vascular endothelial injury exacerbates coronavirus disease 2019: the role of endothelial glycocalyx protection. Microcirculation. (2021) 28:e12654. 10.1111/micc.12654 32791568 PMC7435519

[20] Zhang Y Liu J Jia W Tian X Jiang P Cheng Z AGEs/RAGE blockade downregulates Endothenin-1 (ET-1), mitigating Human Umbilical Vein Endothelial Cells (HUVEC) injury in deep vein thrombosis (DVT). Bioengineered. (2021) 12:1360–8. 10.1080/21655979.2021.1917980 33896376 PMC8806329

[21] Abdolahipour R Nowrouzi A Khalili MB Meysamie A Ardalani S. Aqueous Cichorium intybus L. seed extract may protect against acute palmitate-induced impairment in cultured human umbilical vein endothelial cells by adjusting the Akt/eNOS pathway, ROS: NO ratio and ET-1 concentration. J Diabetes Metab Disord. (2020) 19:1045–59. 10.1007/s40200-020-00603-3 33520822 PMC7843711

[22] Bilia AR Bergonzi MC Boulos JC Efferth T. Nanocarriers to enhance solubility, bioavailability, and efficacy of artemisinins. World J Tradit Chin Med. (2020) 6:26–38. 10.4103/wjtcm.wjtcm_2_20

[23] Cyr AR Huckaby LV Shiva SS Zuckerbraun BS. Nitric oxide and endothelial dysfunction. Crit Care Clin. (2020) 36:307–21. 10.1016/j.ccc.2019.12.009 32172815 PMC9015729

[24] Fraix A Parisi C Seggio M Sortino S. Nitric oxide photoreleasers with fluorescent reporting. Chemistry. (2021) 27:12714–25. 10.1002/chem.202101662 34143909

[25] Wu J Li Z Yuan W Zhao Y Li J Li Z Changes of endothelin-1 and nitric oxide systems in brain tissue during mild hypothermia in a porcine model of cardiac arrest. Neurocrit Care. (2020) 33:73–81. 10.1007/s12028-019-00855-9 31595393

[26] Torres Crigna A Link B Samec M Giordano FA Kubatka P Golubnitschaja O. Endothelin-1 axes in the framework of predictive, preventive and personalised (3P) medicine. EPMA J. (2021) 12:1–41. 10.1007/s13167-021-00248-z PMC8334338 34367381

[27] Dragic S Momcicevic D Zlojutro B Jandric M Kovacevic T Djajić V Serum levels of nitric oxide and endothelin-1 in vasculopathy managed with hyperbaric oxygen therapy. Clin Hemorheol Microcirc. (2020) 75:233–41. 10.3233/CH-190796 32116239

[28] Xu M Zhou W Chen X Zhou Y He B Tan S. Analysis of the biodegradation performance and biofouling in a halophilic MBBR-MBR to improve the treatment of disinfected saline wastewater. Chemosphere. (2021) 269:128716. 10.1016/j.chemosphere.2020.128716 33121810 PMC7578672

[29] Mao YJ Wu JB Yang ZQ Zhang YH Huang ZJ. Nitric oxide donating anti-glaucoma drugs: advances and prospects. Chin J Nat Med. (2020) 18:275–83. 10.1016/S1875-5364(20)30035-2 32402405

